# Supplementary material for: Epitope–Paratope Interaction of a Neutralizing Human Anti-Hepatitis B Virus PreS1 Antibody That Recognizes the Receptor-Binding Motif
Source: Vaccines (Basel). 2021 Jul 7;9(7):754. doi: 10.3390/vaccines9070754 (PMC8310169; doi:10.3390/vaccines9070754)
Supplement: Supplementary file 1 [file vaccines-09-00754-s001.zip › vaccines-1262380-supplementary.pdf]

vaccines

MDPI

Supplementary Figures

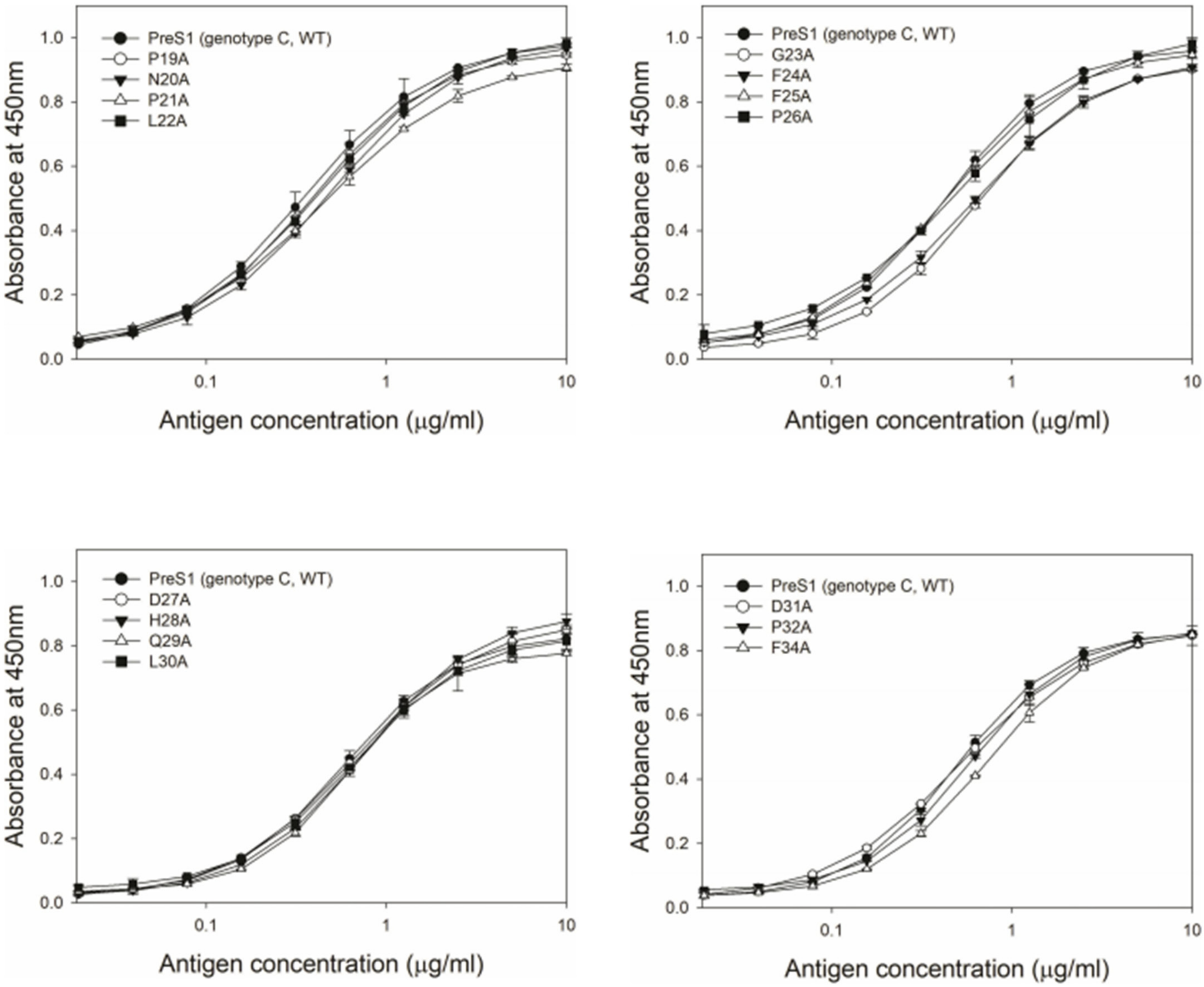

Figure S1. Quantitative ELISA of the antibody-binding activities of purified GST-preS1(aa 1–56)-strep carrying alanine substitution.

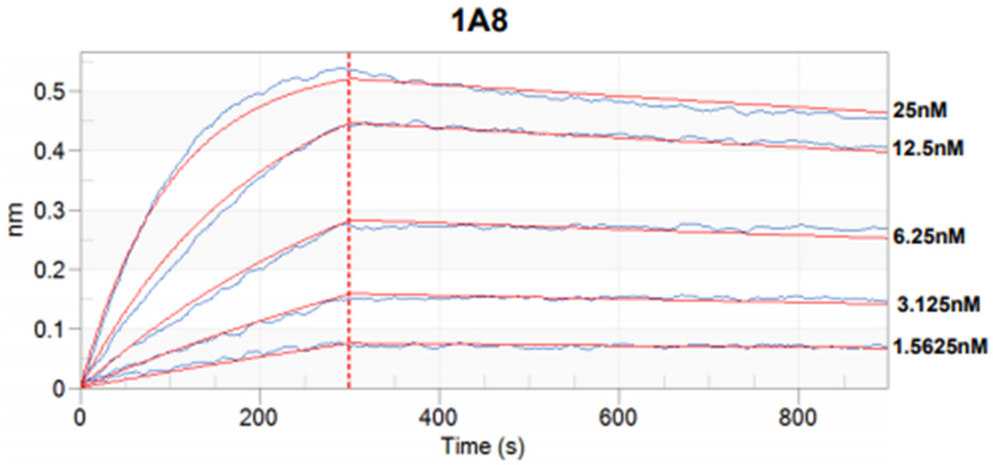

Figure S2. Affinity determination of 1A8 by Bio-Layer interferometry using Octet 384.
